# Replication in Human Intestinal Enteroids of Infectious Norovirus from Vomit Samples

**DOI:** 10.3201/eid2708.210011

**Published:** 2021-08

**Authors:** Marie Hagbom, Jenny Lin, Tina Falkeborn, Lena Serrander, Jan Albert, Johan Nordgren, Sumit Sharma

**Affiliations:** Linköping University, Linköping, Sweden (M. Hagbom, J. Lin, L. Serrander, J. Nordgren, S. Sharma);; Linköping University Hospital, Linköping (T. Falkeborn, L. Serrander);; Karolinska University Hospital, Stockholm, Sweden (J. Albert);; Karolinska Institutet, Stockholm (J. Albert)

**Keywords:** norovirus, infectivity, vomit, human intestinal enteroids, Norwalk virus, viruses, Sweden, enteric infections

## Abstract

A typical clinical symptom of human norovirus infection is projectile vomiting. Although norovirus RNA and viral particles have been detected in vomitus, infectivity has not yet been reported. We detected replication-competent norovirus in 25% of vomit samples with a 13-fold to 714-fold increase in genomic equivalents, confirming infectious norovirus.

Human noroviruses are positive-sense RNA viruses that cause nearly 685 million cases of acute gastroenteritis worldwide per year, including ≈200 million cases in children, resulting in 50,000 child deaths (*1*). The disease is a substantial burden to healthcare systems and carries a global economic cost of ≈US $65 billion each year (*2*). Noroviruses are shed and usually transmitted through the fecal–oral route. However, outbreak investigations have suggested vomiting is a major contributor to transmission; norovirus has been detected in vomitus (*3*–*5*) and oral mouthwash samples (*6*). Despite this documented role in transmission, data on viral loads are limited, and information about infectivity in vomit is lacking (*3*–*5*,*7*).

A Norwalk virus (genus *Norovirus*) human challenge trial found that 56% of vomit samples contained detectable virus, and the median titer was 4.1 × 10^4^ genomic equivalents (GEq)/mL (*7*). Another study reported that nearly half of the participants suffered vomiting postchallenge and on average shed up to 8.0 × 10^5^ GEq/mL in vomit for the Norwalk virus and 3.9 × 10^4^ GEq/mL in vomit for the 2 GII strains studied (*4*). The presence of intact virions in vomit was also reported in an early human challenge study with the Norwalk virus (*8*). These intact virions were detected by immune electron microscopy in concentrated vomit from 1 of the 5 challenge volunteers. These studies indicate that vomit could be a source of major spread of noroviruses, but the presence of infectious virus in vomit has not been reported.

## The Study

To determine the presence of infectious virus in vomit, we used the human intestinal enteroid (HIE) culture system to culture vomit samples positive for norovirus. The system was previously used to replicate human noroviruses from fecal samples (*9*). HIE cultures were established using biopsy specimens from patients who underwent gastric bypass (ethics permission no. 2019–00600, Linköping Ethical Board, Linköping, Sweden). Written informed consent was obtained from all participants. We obtained 28 PCR-positive norovirus vomit samples collected for routine diagnosis from persons with acute gastroenteritis from Karolinska University Hospital (Stockholm, Sweden) and University Hospital of Linköping. The vomit samples were anonymized when received, and only information regarding the initial cycle threshold (C_t_) value was provided. Decoded clinical samples without person-related data and traceability that have not been taken for research purposes do not require ethics or legal clearance according to The Swedish Ethics Review Authority.

The norovirus C_t_ values in the diagnostic PCRs ranged from 13.4 to 31.7. A previous study using fecal samples observed that the replication rate dropped substantially when 1.9 × 10^3^ GEq/mL were used as inoculum for infection (*10*), whereas another study reported loss of infectivity at higher C_t_ values (*11*). Of 28 vomit samples, 20 that had C_t_ values of <26 had 8.9 × 10^6^ to 1.6 × 10^10^ GEq/mL (Table); the remaining 8 vomit samples had <1 × 10^6^ GEq/mL (in undiluted vomit) and were excluded from further evaluation.

**Table:** Details of the norovirus genotypes and titers in the 20 vomit samples tested for norovirus infectivity in human intestinal enteroids*

**Sample name**	Genotype†	Titer, GEq/mL‡
**V1**	GII.4§	7.86 × 10^9^
**V2**	GII.4	1.36 × 10^9^
V3	GII.4	5.28 × 10^8^
V5	GII.4	1.44 × 10^8^
V6	GII.2	8.55 × 10^7^
**V8**	GII.4	1.16 × 10^8^
V11	GII.17	1.21 × 10^8^
V12	GII.4	1.25 × 10^7^
V18	GII.2	5.41 × 10^7^
V19	GII.4	2.00 × 10^8^
V20	GII.2	8.73 × 10^8^
V21	GII.4	5.92 × 10^7^
V22	GII.4	1.91 × 10^7^
V23	GII.4	2.83 × 10^8^
V24	GII.4	1.66 × 10^9^
**V25**	GII.4	1.61 × 10^10^
V29	GII.4	1.51 × 10^8^
V30	GII.4	8.91 × 10^6^
**V32**	GII.4	9.55 × 10^7^
V33	GII.4	3.52 × 10^8^

*Bold indicates vomit samples that showed successful norovirus replication. GEq, genomic equivalent. †Norovirus genotype determined by partial sequencing of the VP1 gene encoding the major capsid protein. ‡Norovirus titer (GEq/mL) in undiluted vomit used to infect human intestinal enteroids. 100µL of 1:100 diluted sample was used as inoculum. **§All GII.4 norovirus detected belonged to the GII.4 Sydney 2012 variant. **

Infectivity was tested on 5-day-old differentiated HIEs established from the jejunum of persons who had undergone gastric bypass surgery. Initial screening to determine infectivity of vomit samples was done with 2 different HIEs (HIE 003 and HIE 004) isolated from secretor-positive persons (i.e., having a functional fucosyltransferase 2 gene). Both HIEs showed similar replication for the same 5 vomit samples. Next, we used HIE 003 for infection in triplicates with 2 technical repeats during quantitative reverse transcription PCR (qRT-PCR) (Figure). Norovirus genotypes in the vomit samples were determined by nucleotide sequencing. We defined infection as a >10-fold increase in GEq 72 hours postinfection (hpi) compared with 2 hpi, determined by qRT-PCR. We compiled details regarding the qRT-PCR method and the isolation, culturing, genotyping or phenotyping, and infection of HIEs (Appendix).

**Figure:**
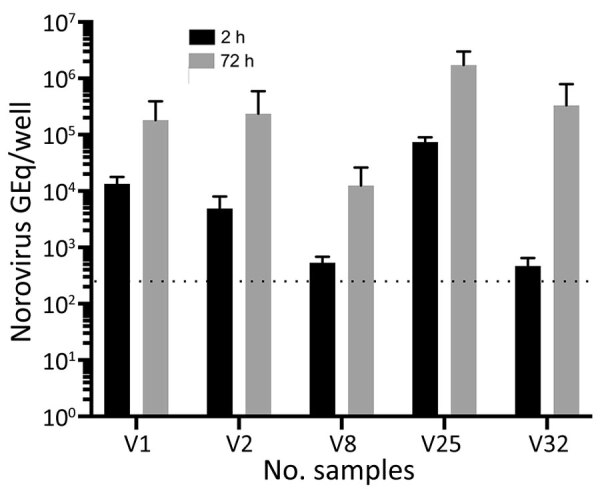
Replication in human intestinal enteroids of norovirus from vomit samples. Differentiated human intestinal enteroid monolayers were inoculated with norovirus-positive vomit samples. The number of norovirus GEq was quantified by reverse transcription quantitative PCR, 2 hours and 72 hours postinfection. Five of 20 vomit samples showed viral replication (defined as a >10-fold increase in the GEq). Data are presented as the mean +SD of biologic triplicates. The inoculum for vomit samples that demonstrated viral replication were as follows: V1, 7.9 × 10^6^ GEq/well; V2, 1.4 × 10^6^ GEq/well; V8, 1.2 × 10^5^ GEq/well; V25, 1.6 × 10^7^ GEq/well; and V32, 9.6 × 10^4^ GEq/well. The dotted lines represent quantitative reverse transcription PCR limit of detection. GEq, genomic equivalent.

Partial nucleotide sequencing of the norovirus capsid region showed that 16 of the 20 vomit samples contained GII.4 norovirus genotype (belonging to GII.4 genotype Sydney 2012 variant), 3 contained GII.2, and 1 contained GII.17 norovirus genotype (Table). In the HIE infectivity assay, 5 of the vomit samples resulted in an increase in GEq, ranging from 13-fold to 714-fold at 72 hpi compared with 2 hpi; all these samples contained GII.4 noroviruses (Figure). The percentage of vomit samples (31.2%) containing GII.4 norovirus that successfully replicated is similar to that reported by Constantini et al. (*10*) using fecal samples positive for norovirus by PCR (25.6%). Of the 4 vomit samples containing GII.2 (n = 3) and GII.17 (n = 1), none demonstrated any replication in HIE, despite 2 GII.2 and 1 GII.17 vomit samples having similar or higher GEq in the inoculum compared to the fecal samples that could be successfully replicated in Constantini et al. (*10*). Of note, this finding might be because of the small number of GII.2-containing vomit samples and GII.17-containing vomit samples tested; not all fecal samples with high viral loads can be successfully replicated (*10*).

## Conclusions

A previous study reported that fecal suspensions that showed successful norovirus replication in HIE cultures contained 1.9 × 10^3^ to 1.7 × 10^7^ GEq in the inoculum, regardless of genogroup or genotype (*10*). In our study, the GII.4 norovirus that could be successfully replicated contained a similar viral load (9.55 × 10^4^ to 1.61 × 10^7^ GEq) in the inoculum used for infection. Vomit samples that failed to show norovirus replication had of 8.91 × 10^3^ to 1.66 × 10^6^ GEq in the inoculum used for infection (Table), which suggests that viral load is not the sole criterion for successful infection in HIEs, as has been reported for norovirus cultured from feces (*10*). Because the vomit samples in this study were anonymized, no information beside the initial norovirus C_t_ value was available. Factors such as long-term storage (*12*) and the time of collection postinfection (*13*) might affect infectivity and cannot be ruled out. Repeated freeze-thaw cycles could also influence the infectivity of viruses, possibly because of the disruption of the capsid proteins, which could degrade the viral genome. However, Richards et al. (*12*) reported that norovirus capsid integrity is not compromised after repeated freeze-thaw cycles. Therefore, despite not knowing the exact long-term storage conditions in the 2 hospitals that provided the vomit samples (although most were stored at −70°C for <3 years), variation in infectivity should not have been caused by multiple freeze-thaw cycles. The time of sample collection also might influence infectivity. Samples should be collected within the first 24 hours after symptom onset. Norovirus can be shed in feces for >7 days, but no studies report infectivity after the initial 48–72 hours after symptom onset (*13*). Although qRT-PCR is standard for detecting norovirus RNA, it does not distinguish infectious virus particles from noninfectious virus particles (*10*).

Although an estimate of the 50% human infectious dose (HID_50_) in vomit containing virus is unknown, it has been calculated to be ≈2,800 GEq for secretor-positive persons challenged with the Norwalk virus (*7*). Comparing the RNA levels in vomit and feces (on the basis of human challenge studies with the Norwalk virus), it was estimated that 1 mL of vomitus contained up to 9,000 HID_50_ of virus (*7*). The combination of a low infectious dose and a large quantity of virus in vomit led to the suggestion that each vomiting event has the potential to infect >150,000 persons (*4*). In our study, we found that >95,500 GEq per inoculum was sufficient for infection of HIEs. Considering the different models studied (human vs. in vitro), the use of strains from different genogroups, and fecal versus vomit inoculum, the similarity in infectious dose is noteworthy.

In conclusion, this study demonstrates that norovirus contained in vomit is infectious. Aerosols and droplets from vomiting could be a source of norovirus transmission.

Appendix Additional information about replication in human intestinal enteroids of infectious norovirus from vomit samples
