# Supplementary material for: Replication in Human Intestinal Enteroids of Infectious Norovirus from Vomit Samples
Source: Emerg Infect Dis. 2021 Aug;27(8):2212–4. doi: 10.3201/eid2708.210011 (PMC8314841; doi:10.3201/eid2708.210011)
Supplement: Appendix — Additional information about replication in human intestinal enteroids of infectious norovirus from vomit samples [file 21-0011-Techapp-s1.pdf]

Article DOI: https://doi.org/10.3201/eid2708.210011

Replication in Human Intestinal Enteroids of Infectious Norovirus from Vomit Samples

Appendix

Materials and Methods

Isolation, Maintenance and Differentiation of Human Intestinal Enteroids

Human intestinal enteroid (HIE) cultures were established by isolating intestinal stem cells from the jejunum section of the small intestine by using biopsy specimens from patients who underwent gastric bypass (ethical permission no. 2019–00600, Linköping Ethical Board, Sweden). Written informed consent was obtained from all participants. The intestinal crypts were isolated and the stem cells were maintained in 3D-Matrigel domes as described previously (*1*) with minor modifications. Differentiation of HIEs in monolayers for infection studies was performed as described previously (*1*).

Infection of HIEs with Norovirus Samples

Vomit samples were diluted 1:100 in GIBCO Advanced DMEM/F12 (ThermoFisher Scientific, https://www.thermofisher.com), vortexed, and centrifuged at 13000 × *g* for 5 min to remove debris. Before infecting, the 5-days differentiated HIE monolayers were washed twice with GIBCO Advanced DMEM/F12. Then, 100 µL of inoculum containing 500 µM glycochenodeoxycholic acid were added onto the cells for 2 h and incubated at 37°C and 5% CO_2. After 2 h, the monolayers were washed twice, before incubating the cells in 100 µL DM supplemented with 500 µM glycochenodeoxycholic acid at 37°C and 5% CO_2 for 72 h.

Determination of Secretor Phenotype by α1,2 Fucose Staining

To determine the secretor phenotype of the HIEs used for infection, fluorescein isothiocyanate–conjugated *Ulex europaeus* Agglutinin I (UEA-I) (Sigma Aldrich, https://www.sigmaaldrich.com), which has α1,2-fucose binding specificity, was used. In brief, the HIE monolayers were fixed with 4% formaldehyde for 1 h at

RT, then washed twice with phosphate-buffered saline. Next, the cells were incubated with fluorescein isothiocyanate–conjugated UEA-I (1:100 dilution, 1 mg/mL) for 1 h at RT in the dark. The cells were then washed twice and incubated with 4,6-diamidino-2-phenylindole (DAPI) for 5 min, washed twice with phosphate-buffered saline, and observed by using a DMi8 fluorescence microscope (Leica Microsystems, https://www.leica-microsystems.com/).

Genotypic Characterization of FUT2 Gene

Genomic DNA was isolated from the HIEs by using QIAGEN QIAamp DNA Kit as per manufacturer's instructions (QIAGEN, https://www.qiagen.com). Region of the exon 2 of the FUT2 gene spanning the G428A mutation was amplified as described previously (*2*). Sanger sequencing of the amplified products was performed by Macrogen (https://dna.macrogen-europe.com).

Viral RNA Extraction

Viral RNA was extracted by using the QIAGEN QIAamp Viral RNA Mini Kit (QIAGEN). After 2 h or 72 h of norovirus infection, enteroids were lysed with AVL lysis buffer and the remaining steps for RNA extraction were performed per the kit manufacturer's instructions. The eluted RNA was used for cDNA synthesis.

cDNA Synthesis

cDNA synthesis was performed by using the iScript cDNA Synthesis Kit (Bio-Rad, https://www.bio-rad.com). Each reaction contained 2 µL of 5× iScript Reaction Mix, 0.5 µL of iScript reverse transcriptase, 2.5 µL of nuclease-free MilliQ H_2O, and 5 µL of extracted viral RNA. The reaction mix was subjected to a priming step at 25°C for 5 min, followed by a reverse transcription step at 46°C for 40 min and an enzyme inactivation step at 95°C for 1 min. The cDNA was either directly used for norovirus quantitative PCR (q-PCR) or stored at −20°C.

Determining Norovirus Replication Using q-PCR

For analysis of viral replication, q-PCR was used to compare virus genomic equivalents at 2 and 72 hours postinfection (hpi). The q-PCR was performed by using TaqMan Universal Probes Supermix, 400 nM NVGIIF (CGYTGGATGCGNTTCCATGA) primer, 400 nM NVGIIR primer (GTCCTTAGACGCCATCATC), and 200 nM GII Probe ([HEX]TGGGAGGGCGATCGCAATCT[BHQ1]), as previously described (*3*–*5*).

The initial denaturation step at 95°C for 3 min was followed by 45 cycles of 95°C for 5 s and 60°C for 30 s. Plasmids containing norovirus GII genogroup gene segment were used as standards to estimate genomic equivalents.

Genotyping Norovirus Samples

To determine norovirus genotypes in vomit samples, a seminested PCR was performed to amplify a region encoding the major capsid protein, VP1. For the first round of amplification, the primers NVGIIF and G2SKR (CCRCCNGCATRHCCRTTRTACAT) were used. In the second round, G2SKF (CNTGGGAGGGCGATCGCAA) and G2SKR were used to amplify the PCR product of the first round (*6*). Both PCR runs consisted of an initial denaturation step at 94°C for 4 min and 40 cycles of 94°C for 30 s, 50°C for 30 s, and 72°C for 1 min, followed by a final elongation at 72°C for 7 min. Sanger sequencing of the amplified products was performed by Macrogen. A web-based open-access norovirus automated genotyping tool was used to assign the norovirus genotypes (*7*). The nucleotide accession numbers are MW419854–MW419873.

References

1. Ettayebi K, Crawford SE, Murakami K, Broughman JR, Karandikar U, Tenge VR, et al. Replication of human noroviruses in stem cell-derived human enteroids. Science. 2016;353:1387–93. PubMed https://doi.org/10.1126/science.aaf5211

2. Piedade J, Nordgren J, Esteves F, Esteves A, Teodósio R, Svensson L, et al. Molecular epidemiology and host genetics of norovirus and rotavirus infections in Portuguese elderly living in aged care homes. J Med Virol. 2019;91:1014–21. PubMed https://doi.org/10.1002/jmv.25425

3. Kageyama T, Kojima S, Shinohara M, Uchida K, Fukushi S, Hoshino FB, et al. Broadly reactive and highly sensitive assay for Norwalk-like viruses based on real-time quantitative reverse transcription-PCR. J Clin Microbiol. 2003;41:1548–57. PubMed https://doi.org/10.1128/JCM.41.4.1548-1557.2003

4. Oluwatoyin Japhet M, Adeyemi Adesina O, Famurewa O, Svensson L, Nordgren J. Molecular epidemiology of rotavirus and norovirus in Ile-Ife, Nigeria: high prevalence of G12P[8] rotavirus strains and detection of a rare norovirus genotype. J Med Virol. 2012;84:1489–96. PubMed https://doi.org/10.1002/jmv.23343

5. Nordgren J, Bucardo F, Dienus O, Svensson L, Lindgren PE. Novel light-upon-extension real-time PCR assays for detection and quantification of genogroup I and II

noroviruses in clinical specimens. J Clin Microbiol. 2008;46:164–70. PubMed https://doi.org/10.1128/JCM.01316-07

6. Kojima S, Kageyama T, Fukushi S, Hoshino FB, Shinohara M, Uchida K, et al. Genogroup-specific PCR primers for detection of Norwalk-like viruses. J Virol Methods. 2002;100:107–14. PubMed https://doi.org/10.1016/S0166-0934(01)00404-9

7. Kroneman A, Vennema H, Deforche K, v d Avoort H, Peñaranda S, Oberste MS, et al. An automated genotyping tool for enteroviruses and noroviruses. J Clin Virol. 2011;51:121–5. PubMed https://doi.org/10.1016/j.jcv.2011.03.006
